# High activity catechol 1,2-dioxygenase from *Stenotrophomonas maltophilia* strain KB2 as a useful tool in *cis*,*cis*-muconic acid production

**DOI:** 10.1007/s10482-013-9910-8

**Published:** 2013-03-28

**Authors:** Urszula Guzik, Katarzyna Hupert-Kocurek, Małgorzata Sitnik, Danuta Wojcieszyńska

**Affiliations:** Department of Biochemistry, Faculty of Biology and Environmental Protection, University of Silesia in Katowice, Jagiellonska 28, 40-032 Katowice, Poland

**Keywords:** *cis*,*cis*-muconic acid production, *Stenotrophomonas*, Catechol 1,2-dioxygenase, Substrate specificity, Kinetic

## Abstract

This is the first report of a catechol 1,2-dioxygenase from *Stenotrophomonas maltophilia* strain KB2 with high activity against catechol and its methyl derivatives. This enzyme was maximally active at pH 8.0 and 40 °C and the half-life of the enzyme at this temperature was 3 h. Kinetic studies showed that the value of *K*_m_ and *V*_max_ was 12.8 μM and 1,218.8 U/mg of protein, respectively. During our studies on kinetic properties of the catechol 1,2-dioxygenase we observed substrate inhibition at >80 μM. The nucleotide sequence of the gene encoding the *S. maltophilia* strain KB2 catechol 1,2-dioxygenase has high identity with other *cat*A genes from members of the genus *Pseudomonas*. The deduced 314-residue sequence of the enzyme corresponds to a protein of molecular mass 34.5 kDa. This enzyme was inhibited by competitive inhibitors (phenol derivatives) only by ca. 30 %. High tolerance against condition changes is desirable in industrial processes. Our data suggest that this enzyme could be of use as a tool in production of *cis*,*cis*-muconic acid and its derivatives.

## Introduction

Adipic acid is an important industrial compound, production of which is necessary for the manufacture of nylon, polyurethane, insecticides and bactericides. One of the most important intermediates during synthesis of this acid is *cis*,*cis*-muconic acid, which easily converts to adipic acid by hydrogenation (Wu et al. 2006). Moreover, *cis*,*cis*-muconic acid has reactive carboxylate groups and a configuration of conjugated double bonds that can be useful as a raw material for new functional resins, pharmaceuticals and agrochemicals (Bang et al. 1996; Wingard and Finn 1969). However, there have been no secure and economical methods to synthesize *cis*,*cis*-muconic acid. Several reports have described the preparation of this compound starting with straight-chain compounds, however, the product has been the more thermodynamically stable *trans*,*trans* isomer and therefore in recent years biological methods have been used more often to produce *cis*,*cis*-muconic acid (Wingard and Finn 1969). For example, Schmidt and Knackmuss (1984) described *Pseudomonas* sp. strain B13, which produced *cis*,*cis*-muconate and 2-fluoro-*cis*,*cis*-muconate from benzoate and 3-fluorobenzoate. Kaneko et al. (2011) used recombinant *Escherichia coli* cells expressing the *catA* gene for high-yield production of *cis*,*cis*-muconic acid from catechol. This gene codes for catechol 1,2-dioxygenase (1,2-CTD), which plays a key role in catalysing aromatic ring cleavage at the *ortho* position to yield *cis*,*cis*-muconic acid.

Recently several catechol 1,2-dioxygenases, generally from members of the genera *Arthrobacter*, *Acinetobacter*, *Pseudomonas*, *Sphingomonas* and *Rhodococcus* have been described (Earhart et al. 2005; Guo et al. 2009; Guzik et al. 2011; Kim et al. 1998; Matera et al. 2010; Matsumura et al. 2004; Melo et al. 2010; Murakami et al. 1998; Nadaf and Ghosh 2011; Saxena and Thakur 2005; Wang et al. 2006). Most of them are enzymes with molecular mass 30–34 kDa, which consist of two either identical or non-identical subunits (Bruijnincx et al. 2008; Bugg 2003; Guzik et al. 2011; Patel et al. 1976; Vaillancourt et al. 2006). At the dimeric interface is located a hydrophobic cavity which is connected to phospholipid molecules (Matera et al. 2010). Nonheme iron in the ferric state is used as a cofactor for intradiol dioxygenases (Bruijnincx et al. 2008; Bugg 2003; Guzik et al. 2011; Patel et al. 1976; Vaillancourt et al. 2006). The iron is ligated by two histidines and two tyrosines. The initial coordination geometry is trigonal bipyramidal with tyrosine, histidine and a bound hydroxyl in the equatorial plane, and the other tyrosine and histidine as axial ligands (Earhart et al. 2005). The catalytic cycle of the intradiol dioxygenases involves binding of the catechol as a dianion, binding of dioxygen to the metal, in sequence formation of a peroxo and hydroperoxo intermediate. In the next step, the Criegee rearrangement occurs and O–O bond cleavage, which involves acyl migration to yield the cyclic anhydride and an iron-bound oxide or hydroxide, takes place. Hydrolysis of the anhydride leads to the formation of the final acyclic product (Bugg 2003; Bugg and Lin 2001; Vaillancourt et al. 2006; Vetting and Ohlendorf 2000).

Comprehensive studies on the substrate diversity and catalytic properties of catechol 1,2-dioxygenases are essential to facilitate the cheap and safe synthesis of *cis*,*cis*-muconic acid. Now we report novel a catechol 1,2-dioxygenase, characterized by high activity, isolated from *Stenotrophomonas maltophilia* strain KB2 which converted benzoic acid to *cis*,*cis*–muconic acid. We have identified the gene encoding the catechol 1,2-dioxygenase in *S. maltophilia* KB2 and deduced a putative three-dimensional structure of this enzyme from the amino acid sequence.

## Materials and methods

### Media and culture conditions

*Stenotrophomonas maltophilia* KB2 (VTT E-113197) was cultivated in mineral salts medium (MSM), as described previously (Wojcieszyńska et al. 2011) in the presence of 6 mM benzoic acid. Cultures were incubated at 30 °C and agitated at 130 rpm.

### Preparation of cell extracts

Cells were harvested in the late exponential growth phase and centrifuged at 4,500×*g* for 15 min at 4 °C. Next, the cells were washed with 50 mM phosphate buffer, pH 7.0, and resuspended in the same buffer. Cells were sonicated 6× for 15 s and centrifuged at 9,000×*g* for 30 min at 4 °C. The supernatant was used as crude extract for enzyme assays.

### Enzyme assays

Benzoic acid was used as the inducer of catechol 1,2-dioxygenase in the growth medium. Enzymatic activity of the enzyme was measured spectrophotometrically (Wojcieszyńska et al. 2011). After the addition of the enzyme, vials were incubated at 35 °C in a water-bath with shaking. At specific time intervals, 1 ml aliquots were withdrawn and used to monitor the reaction progress by measuring the product *cis*,*cis*-muconic acid at 260 nm. The extinction coefficient of the oxidation product of catechol was determined as ε_260 nm_ = 16,800/M cm. One unit of enzyme activity was defined as the amount of enzyme required to generate 1 μmol of product per minute at 35 °C. The protein concentration was determined by the dye-binding procedure of Bradford using bovine serum albumin as a standard (Bradford 1976).

### pH and temperature optima of catechol 1,2-dioxygenase

The effect of pH on the enzyme activity was determined by measuring the activity at 35 °C over the pH range 2.2–12.0 using the following buffers: 0.05 M glycine (pH 2.2), 0.05 M phosphate-citrate (pH 3.0–5.0), 0.05 M Sörensen (pH 6.0–8.0), 0.05 M borate (pH 9.0–10.0), and 0.05 Britton-Robinson (pH 11.00–12.00).

The optimum temperature was determined by assaying the enzyme activity at various temperatures (4–60 °C) in 50 mM phosphate buffer solution (pH 7.2).

### Determination of kinetic constants of catechol 1,2-dioxygenase

The catalytic parameters (Michaelis–Menten constant, *K*_m_, and Maximum velocity, *V*_max_) for the enzyme were calculated by measuring the initial linear rates of the enzymatic reaction after the addition of different concentrations of catechol ranging from 0 to 100 μM at 35 °C. Three independent measurements were carried out for each substrate concentration. *K*_m_ and *V*_max_ were calculated from the Michaelis–Menten equation.

### Substrate specificity

The impact of various substituted derivatives of aromatic compounds on enzyme activity was evaluated by incubating the enzyme with the respective aromatic compound (at 1 mM) for 3 min and assaying the activity. Dihydroxy-substituted derivatives of arene studied were 3- and 4-methylcatechol, 4,5- and 3,5-dichlorocatechol, and hydroquinone. The molar extinction coefficient used for the product from hydroquinone was 11,000/M cm (at 320 nm) (Kolvenbach et al. 2011; Spain and Gibson 1991). Catechol 1,2-dioxygenase activities for chlorinated and methylated derivatives of catechol were determined by the procedures of Dorn and Knackmuss (1978).

### Activity in the presence of inhibitors

The impact of various phenols and chelators on enzyme activity was evaluated by incubating the enzyme with the respective inhibitor for 3 min and then assaying the residual activity. At specific time intervals, 1 ml aliquots were withdrawn and used to monitor the reaction progress by measuring the product *cis*,*cis*-muconic acid. Assay of catechol 1,2-dioxygenase was performed in the same way as in the case of non-inhibited enzyme. The phenols studied were 2-methylphenol, 3-methylphenol, 4-methylphenol, 2-chlorophenol, 4-chlorophenol, 2,4-dichlorophenol, each at 0.1, 0.2, and 0.3 mM concentration. Chelators studied were phenanthroline, EDTA, and 2,2′-dipirydyl (each at 1, 2, and 3 mM).

### Amplification of catechol 1,2-dioxygenase gene

Genomic DNA, plasmid DNA isolation and other DNA manipulations were carried out according to established procedures (Sambrook et al. 1989). Oligonucleotides for the PCR were purchased from IBB PAN (Warsaw, Poland). Detection of the 1,2-CTD gene was carried out with primer designed by Guzik et al. 2011 i.e. 1,2D_zewF (GATGGATCCGTGAAAATTTCCCACATGC) and 1,2D_zewR (TGGATCCAGTAACGTTGCAGGTGCT). The PCR mixtures contained 0.5 μM of each primer, 10× *Pfu* buffer + MgSO_4_ (2 mM Mg^2+^), 10 mM K^+^, 3 % DMSO, 0.2 mM of each deoxynucleoside triphosphate, 1.25 U *Pfu* DNA polymerase (Sigma) and plasmid or chromosomal DNA as a template. For the 1,2-CTD genes, the annealing temperature was 61 °C (30 s) in the first 10 cycles followed by a step down to 59 °C (30 s) in the next 15 cycles, and 57 °C (30 s) in the last 15 cycles. Aliquots (10 μl) of the PCR products were analyzed by electrophoresis on a 1.0 % agarose gel stained with 0.5 ug/ml ethidium bromide. Gene sequencing was performed by using a Big Dye^R^ Terminator Cycle Sequencing Kit (Applied Biosystem) and AbiPrism^®^3100 Genetic Analyzer. Computer analysis and processing of sequence information were performed by using Chromas LITE software (Technelysium Pty, Tewantin, Australia). The nucleotide sequence obtained for the catechol 1,2-dioxygenase gene from *S. maltophilia* strain KB2 has been deposited in the NCBI GenBank database under the accession number EU000397.1.

### Molecular modeling of the catechol 1,2-dioxygenase enzyme

The amino acid sequence of the catechol 1,2-dioxygenase was deduced and followed by multiple sequence alignment using the CLC Free Workbench 6.3 software. The deduced structure of the catechol 1,2-dioxygenase was modeled using the interactive mode of the 3D-JIGSAW protein comparative modeling server (http://bmm.cancerresearchuk.org/~3djigsaw/). Structure models as x.*pdb* data files were analyzed using RasMol 2.6 software package.

## Results and discussion

### Production of *cis*,*cis*-muconic acid by catechol 1,2-dioxygenase

**Fig. 1:**
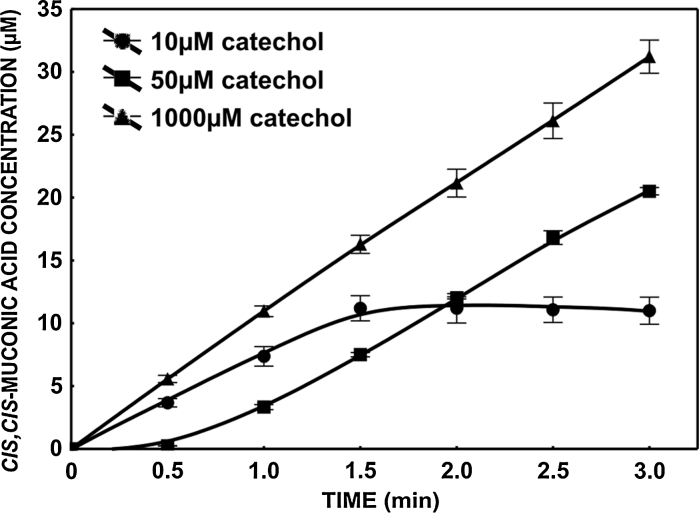
Rate of *cis*,*cis*-muconic acid formation from various catechol concentrations (amount of used protein 7 μg)

In last few decades enzymes with potential for usage in chemical synthesis at the industrial scale have been sought. It is especially important for production of stereoisomers since enzymes exhibit regioselectivity and stereoselectivity (Ran et al. 2008). Several studies have demonstrated production of *cis*,*cis*-muconic acid by microorganisms from benzene, toluene, benzoic acid or catechol (Bang and Choi 1995; Bang et al. 1996; Frost and Draths 1996, 1997). For example, a mutant strain of *Arthrobacter* sp. produced 44 g/l of *cis*,*cis*-muconic acid in two days of culture (Mizuno and Yoshikawa 1990). For the further enhancement of the *cis*,*cis*-muconic acid productivity, it is necessary to obtain high activity catechol 1,2-dioxygenase, the key enzyme in the *cis*,*cis*-muconate biosynthetic pathway (Kim et al. 1998; Wu et al. 2006). An earlier study showed that catechol 1,2-dioxygenase from *S. maltophilia* KB2 was observed after growing the strain in the presence of benzoate (Wojcieszyńska et al. 2011). We considered that the formation of this compound is dependent on substrate concentration. Figure 1 shows that the rate of *cis*,*cis*-muconic acid synthesis averaged 10 μM/min. The molar conversion yield based on the amount of consumed catechol was the theoretical value of 100 % (mol/mol). Similar results was obtained by Kaneko et al. (2011) during production of *cis*,*cis*-muconic acid by recombinant *E. coli* cells that expressed a *catA* gene from *Pseudomonas putida* mt-2.

### Sequence analysis of the catechol 1,2-dioxygenase gene

**Fig. 2:**
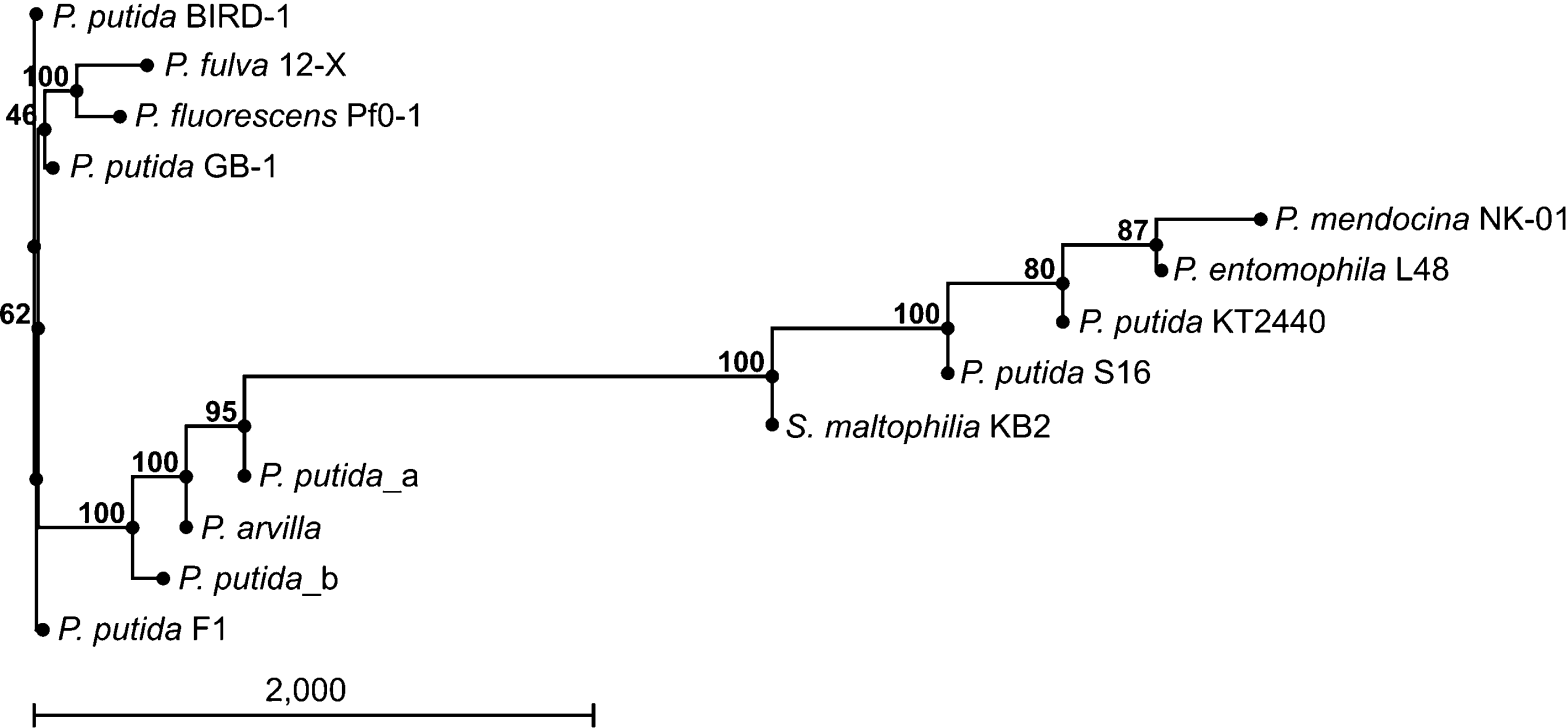
Phylogenetic tree showing the position of the catechol 1,2-dioxygenase sequence from *S. maltophilia* KB2 in comparison with reference catechol 1,2-dioxygenases from other bacterial strains. The numbers at branch points indicate the confidence (in percent) as determined by bootstrap analysis with 100 replicates. The accession codes in the GenBank database and their origins are as follows: CP002290.1 for *Pseudomonas putida* BIRD-1 catechol 1,2-dioxygenase; CP002727.1 for *Pseudomonas fulva* 12-X catechol 1,2-dioxygenase; CP000094.2 for *Pseudomonas fluorescens* Pf0-1 catechol 1,2-dioxygenase; CP000926.1 for *Pseudomonas putida* GB-1 catechol 1,2-dioxygenase; CP002620.1 for *Pseudomonas mendocina* NK-01 catechol 1,2-dioxygenase; CT573326.1 for *Pseudomonas entomophila* L48 catechol 1,2-dioxygenase; AE015451.1 for *Pseudomonas putida* KT2440 catechol 1,2-dioxygenase; EU000397 for *Stenotrophomonas maltophilia* KB2 catechol 1,2-dioxygenase; D37782.1 for *Pseudomonas putida*_a catechol 1,2-dioxygenase; D37783.1 for *Pseudomonas arvilla* catechol 1,2-dioxygenase; AF363241.1 for *Pseudomonas putida*_b catechol 1,2-dioxygenase; CP000712.1 for *Pseudomonas putida* F1 catechol 1,2-dioxygenase

Genes encoding catechol dioxygenases can be located on plasmids or/and on the chromosome (Neidle et al. 1998; Sauret-Ignazi et al. 1996;Vaillancourt et al. 2006; Wojcieszyńska et al. 2011). Our research indicated that strain KB2 contains plasmid DNA (Wojcieszyńska et al. 2011) and we thus performed PCR with chromosomal and plasmid DNA as a template. To amplify the catechol 1,2-dioxygenase *S. maltophilia* KB2 gene we used primers 1,2D_zewF and 1,2D_zewR (Guzik et al. 2011). A PCR product was obtained only with chromosomal DNA as a template, indicating that the gene is located on the chromosomal DNA. Sequencing of the PCR product resulted in a 1243 nucleotide sequence (deposited in GenBank under accession number EU000397). A phylogenetic tree was created (Fig. 2), based upon the catechol 1,2-dioxygenase gene sequences found in GenBank and the new sequence obtained in this study. There was high homology found with other intradiol dioxygenase genes.

### Structural properties of the catechol 1,2-dioxygenase

Knowledge of the catechol 1,2-dioxygenases 3-D structure can provide the important information into the molecular mechanisms of these enzymes. The deduced 314-residue amino acid sequence of *S. maltophilia* KB2 (deposited in the GenBank under accession number ABS86780.1) enzyme corresponds to a protein of molecular mass 34.5 kDa. Similar molecular weights for dioxygenase from *Acinetobacter calcoaceticus* and *P. putida* N6 were obtained by Neidle et al. (1998) and Guzik et al. (2011), respectively.

**Fig. 3:**
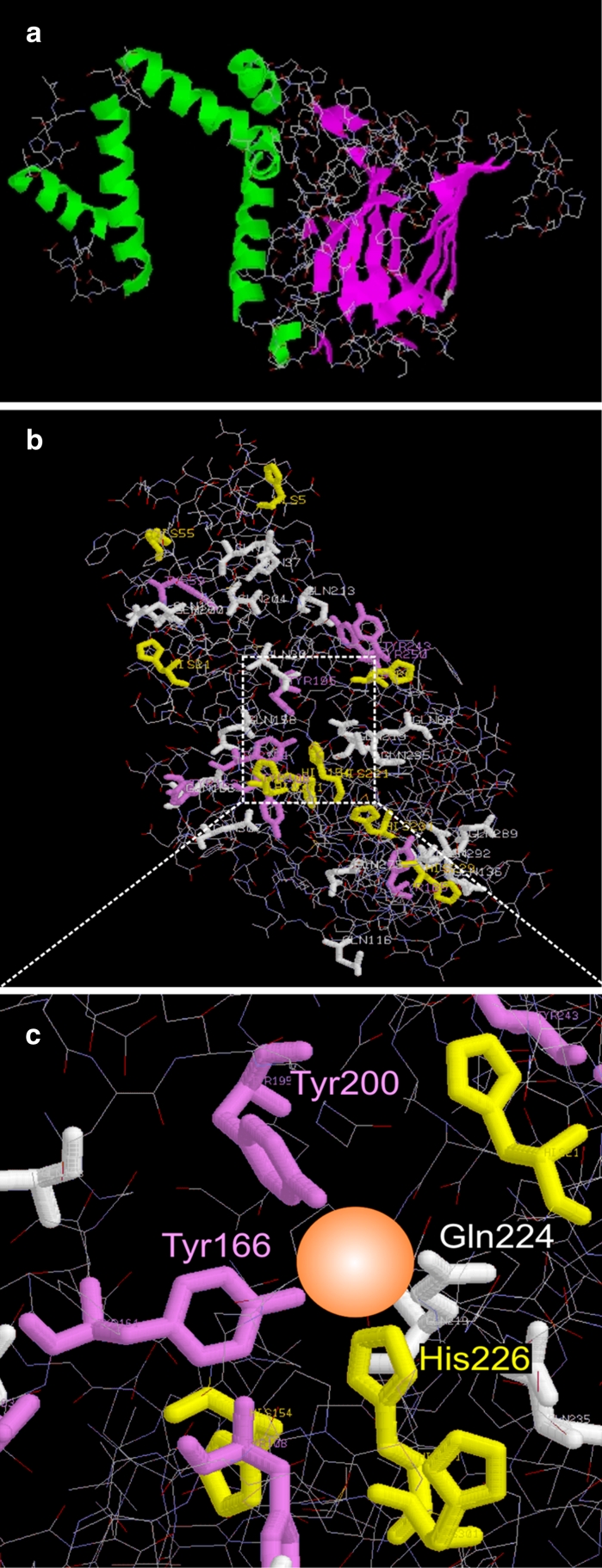
The deduced 3-D structure of the catechol 1,2-dioxygenase from *S. maltophilia* KB2; **a** localization of the molecular domains; **b**, and **c** amino acid residues involved in ferric ions coordination

We predicted the 3-D structure of the catechol 1,2-dioxygenase from strain KB2 based on the deduced amino acid sequence by using the interactive mode of the 3D-JIGSAW protein comparative modeling server. Catechol 1,2-dioxygenase from strain KB2 (Fig. 3a) probably possesses an *N*-terminal domain with five α helices and a C-terminal domain consisting of β-sheets–structures typical for other intradiol dioxygenases as reported previously (Guzik et al. 2011; Ohlendorf et al. 1994; Vaillancourt et al. 1998). Similar molecular structure was also noted in another study of *Pseudomonas arvilla* C-1 catechol 1,2-dioxygenase, catechol 1,2-dioxygenase and 4-chlorocatechol 1,2-dioxygenase from *Rhodococcus opacus* 1CP (Earhart et al. 2005; Ferraroni et al. 2004; Matera et al. 2010). The α helices localized within *N*-terminal domain of the enzyme of strain C-1, like other known intradiol enzymes, were found to be involved in dimerization of enzyme subunits (Bugg 2003; Vaillancourt et al. 2006; Wojcieszyńska et al. 2011).

**Fig. 4:**
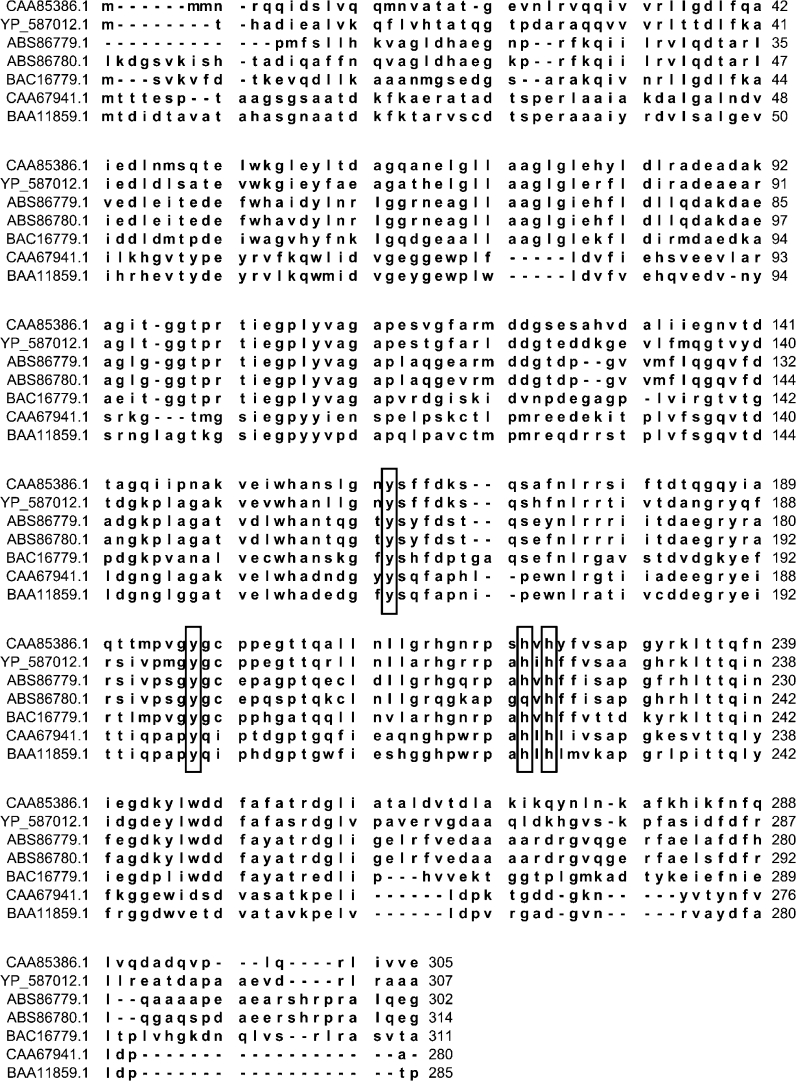
Multiple sequence alignment of predicted catechol 1,2-dioxygenase amino acid sequences carried out using CLC sequence viewer. Aligned sequences are 1,2-CTD of *Acinetobacter calcoaceticus* NCIB8250 (CAA85386.1), 1,2-CTD of *Alcaligenes eutrophus* CH34 (YP_587012.1), 1,2-CDT of *Pseudomonas putida* N6 (ABS86779.1), 1,2-CTD of *Stenotrophomonas maltophilia* KB2 (ABS86780.1), 1,2-CTD of *Burkholderia* sp. TH2 (BAC16779.1), CatA of *Rhodococcus opacus* 1CP (CAA67941.1), and 1,2-CTD of *Rhodococcus erythropolis* AN-13 (BAA11859.1). *Residues in* *solid boxes* indicate the Fe ligands

Intradiol dioxygenases coordinate ferric ion by two histidine, two tyrosine residues and one hydroxyl ion in a trigonalbipyramidal geometry (Bruijnincx et al. 2008; Bugg and Lin 2001; Earhart et al. 2005; Ferraroni et al. 2004; Matera et al. 2010; Melo et al. 2010). Within the active site of the *R. opacus* 1CP 4-chlorocatechol 1,2-dioxygenase, the coordination residues were identified at positions His-194, His-196, Tyr-134, and Tyr-169 (Ferraroni et al. 2004). The catechol 1,2-dioxygenase isolated from this same strain possess as iron ligands: Tyr-162, Tyr-196, His-220, and His-222 (Matera et al. 2010). Our work predicts His-226 Tyr-166, and Tyr-200 to be involved in ferric ions coordination (Figs. 3b, c, 4). However, comparison of the deduced amino acid sequence of the catechol 1,2-dioxygenase from *S. maltophilia* KB2 with other catechol 1,2-dioxygenases has shown that one of conserved active-site residues was altered in the strain KB2 sequence. We predict Gln-224 as a fourth ligand of iron ion (Fig. 4). Displacing one of the key iron bound ligands can cause changes in catalytic properties of enzyme and therefore we examined these in our study.

### Kinetic properties of catechol 1,2-dioxygenase in *S. maltophilia* KB2 cell extracts

**Fig. 5:**
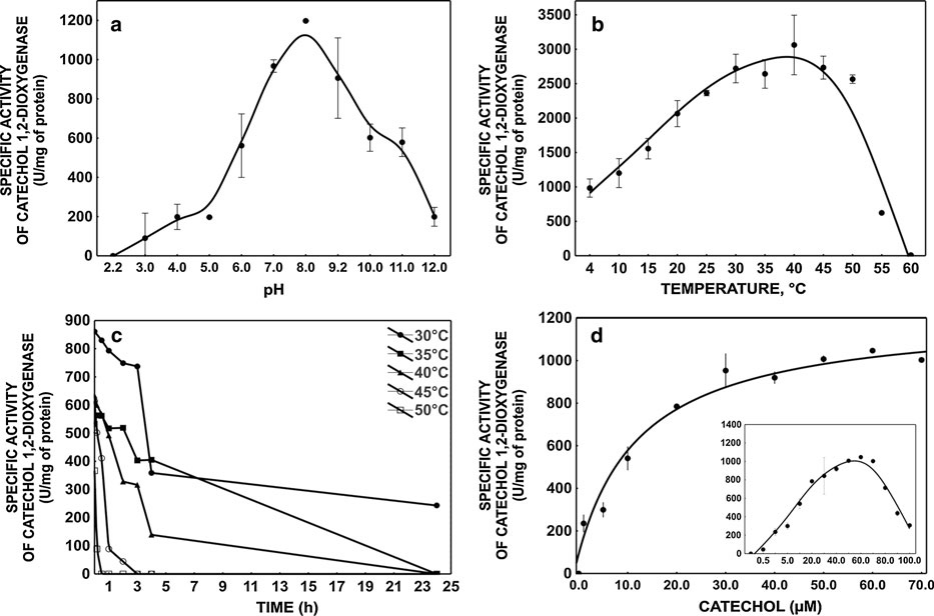
Effects of pH (**a**), temperature (**b**), thermal stability (**c**) and substrate concentration (**d**) on catechol 1,2-dioxygenase activity in *S. maltophilia* KB2 cell extracts. The data points represent the average of 3 independent experiments

The pH-activity and temperature-activity curves showed that the maximum catechol 1,2-dioxygenase activity (3,062 U/mg protein) was at pH 8 and 40 °C, respectively (Fig. 5a, b). On the other hand, the examined enzyme was not very stable in this temperature (Fig. 5c). The half-life of the enzyme at 40 °C was 3 h (Fig. 5b). Interestingly, the enzyme lost 16.5 % of its enzymatic activity at 50 °C and the activity rapidly declined at 55 °C (Fig. 5b). A similar effect was observed by Wang et al. (2006) and Murakami et al. (1998) for catechol 1,2-dioxygenase from *Pseudomonas aeruginosa* and *Arthrobacter* species BA-5-17, respectively. The enzyme isolated from strain KB2 lost 100 % of its original enzymatic activity at pH 2.2 and about 83 % at pH 12.0 (Fig. 5a). The optimal pH of the catechol 1,2-dioxygenase is high compared with that of catechol 1,2-dioxygenase from *Pseudomonas fluorescens,* *P. aeruginosa* and *Rhodococcus* sp. NCIM2891 (Nadaf and Ghosh 2011; Saxena and Thakur 2005; Wang et al. 2006).

In order to calculate values of *K*_m_ and *V*_max_ parameters, the activity of the *S. maltophilia* KB2 catechol 1,2-dioxygenase was measured at different substrate concentrations as detailed in Materials and Methods. The *K*_m_ and *V*_max_ values obtained were 12.18 μM and 1,218.8 U/mg of protein, respectively (Fig. 5d). This *V*_max_ value is notably higher (approximately 20–100-fold) than the activity of other catechol 1,2-dioxygenases. Catechol 1,2-dioxygenase form *Acinetobacter radioresistens* showed 25.8 U/mg of protein (Briganti et al. 2000). Suvorova et al. (2006) and Solyanikova et al. (2009) characterized catechol 1,2-dioxygenase from *R. opacus* 1CP and *R. opacus* 6a with activities of 9.6 U/mg of protein and 55.5 U/mg of protein, respectively. Of note, the *K*_m_ value was 2-fold higher than those obtained by Wang et al. (2006) and Nadaf and Ghosh (2011). This result may therefore indicate lower affinity of enzyme to the substrate.

During our studies on kinetic properties of the catechol 1,2-dioxygenase, substrate inhibition at >80 μM was observed (Fig. 5d). In line with our results, Sauret-Ignazi et al. (1996) observed inhibition activity of an *Alicaligenes eutrophus* CH34 1,2-dioxygenase which catalyses tetrachlorocatechol degradation.

### Influence of various substrates on catechol 1,2-dioxygenase activity in *S. maltophilia* KB2 cell extracts

Differences in substrate specificity is one of the interesting characteristics noted among the isofunctional dioxygenases from various sources. The relative activities of the catechol 1,2-dioxygenase from strain KB2 towards various substrates are given in Table 1. It was found that the enzyme showed activity against catechol, 3-methylcatechol, and 4-methylcatechol. No activity was observed for 3-chloro- and 4-chlorocatechol, 3,5-dichloro- and 4,5-dichlorocatechol and hydroquinone. It could be interpreted that a haloatom might prevent the dioxygenase from attacking the ring. Similar results were obtained by Briganti et al. (2000), Matsumura et al. (2004) and Murakami et al. (1998) for intradiol dioxygenases isolated from *A. radioresistens*, *Rhodococcus* sp. AN-22 and *Arthrobacter* species BA-5-17, respectively. Giedraityte and Kalediene (2009) reported only 27 and 6 % of the relative activity to catechol (7.42 U/mg of protein) of a catechol 1,2-dioxygenase from *Geobacillus* sp. towards 3-methylcatechol and 4-methylcatechol, respectively. Remarkably broader substrate specificity was described by Wang et al. (2006) and Guo et al. (2009) during characterization of catechol 1,2-dioxygenases from *P. aeruginosa* and *Sphingomonas xenophaga* QYY, respectively. The catechol 1,2-dioxygenase from *R. opacus* 1CP showed high activity against to catechol and methylcatechols. Moreover, this enzyme catalyzed cleavage of chlorocatechols, pyrogallol, hydroxyquinol, 2,3- and 3,4-dihydroxybenzoic acid ring (Matera et al. 2010).

**Table 1:** Substrate specificity of catechol 1,2-dioxygenase from *S. maltophilia* KB2

Substrate	Activity of free enzyme, %
Control–catechol	100.0 ± 0.0
3-Methylcatechol	11.6 ± 2.1
4-Methylcatechol	23.0 ± 0.6
3-Chlorocatechol	0.0 ± 0.0
4-Chlorocatechol	0.0 ± 0.0
3,5-Dichlorocatechol	0.0 ± 0.0
4,5-Dichlorocatechol	0.0 ± 0.0
Hydroquinone	0.0 ± 0.0

Data shown represent the average of three independent trials ± standard deviation

### Enzyme activity in *S. maltophilia* KB2 cell extracts in the presence of inhibitors

Phenols substituted in the *ortho* position, which structurally mimic catechols, are known as competitive inhibitors of catechol 1,2-dioxygenases because they coordinate to the iron (III) ion in the enzyme active site (Sauret-Ignazi et al. 1996; Vaillancourt et al. 1998). Most of the phenols studied affected enzyme activity at all tested concentrations (Table 2). Sauret-Ignazi et al. (1996) observed greater sensitivity of catechol 1,2-dioxygenase in the presence of *para* substituted phenols. However the catechol 1,2-dioxygenase from strain KB2 strain did not reveal dependence of activity changes on position and kind of substituents. A similar effect was observed by Kolomytseva et al. (2010) during analysis of the influence of monochloro- and monomethylphenols on activity of chlorocatechol 1,2-dioxygenases from *Rhodococcus opacus* 1CP.

**Table 2:** Effect of phenols and chelators on the activity of catechol 1,2-dioxygenase from *S. maltophilia* KB2

Compound	Concentration (mM)	Relative activity of free enzyme (%)
None		100.0 ± 0.0
2-Methylphenol	0.1	75.3 ± 6.9
	0.2	103.9 ± 4.7
	0.3	82.4 ± 5.4
3-Methylphenol	0.1	79.6 ± 4.3
	0.2	63.7 ± 0.4
	0.3	68.3 ± 2.4
4-Methylphenol	0.1	92.1 ± 6.5
	0.2	70.5 ± 10.2
	0.3	72.5 ± 0.4
2-Chlorophenol	0.1	68.9 ± 3.2
	0.2	67.3 ± 0.8
	0.3	83.0 ± 6.6
4-Chlorophenol	0.1	89.0 ± 6.8
	0.2	87.0 ± 7.9
	0.3	87.0 ± 5.0
2,4-Dichlorophenol	0.1	74.0 ± 3.4
	0.2	94.3 ± 8.1
	0.3	70.2 ± 2.6
Phenanthroline	1	1.0 ± 0.0
	2	0.8 ± 0.1
	3	0.0 ± 0.0
EDTA	1	6.3 ± 0.1
	2	5.7 ± 0.3
	3	0.9 ± 0.2
2,2′-Dipirydyl	1	0.1 ± 0.0
	2	0.0 ± 0.0
	3	0.0 ± 0.0

Data shown represent the average of three independent trials ± standard deviation

The sensitivity of the catechol 1,2-dioxygenase from strain KB to both ferrous and ferric iron chelators (Table 2) may reflect the fact that the iron of the enzyme active site is more weakly bound than in the enzyme from *Arthrobacter* species BA-5-17 (Murakami et al. 1998), *Trichosporon cutaneum* (Varga and Neujahr 1970), *P. aeruginosa* (Wang et al. 2006) *A. calcoaceticus* (Patel et al. 1976), or *Rhodococcus* sp. AN-22 (Matsumura et al. 2004). Varga and Neujahr (1970) suggested a correlation between substrate specificity and the affinity of iron to catechol 1,2-dioxygenases. They reported that dioxygenases with strongly bound iron had narrow substrate specificity and vice versa. Our results are at variance with this suggestions since the catechol 1,2-dioxygenase from strain KB2 has a narrow specificity and apparently weakly bound iron. The sensitivity of our enzyme ton the chelators may be connected with the untypical ligand (Gln-224) of iron in the active site of the dioxygenase (Fig. 3c).

In conclusion catechol 1,2-dioxygenase from *S. maltophilia* strain KB2 could be a useful tool in the production of *cis*,*cis*-muconic acid and its derivatives due to its high activity. The high activity of the enzyme in the presence of methylcatechols may enables it to be used to produce methyl derivatives of *cis*,*cis*-muconic acid. Moreover, the temperature and pH tolerance, and resistance to competitive inhibitors, may be desirable features of the catechol 1,2-dioxygenase from KB2 strain for industrial processes .
